# Lobbying and nutrition policy in Canada: a quantitative descriptive study on stakeholder interactions with government officials in the context of Health Canada’s Healthy Eating Strategy

**DOI:** 10.1186/s12992-022-00842-4

**Published:** 2022-05-26

**Authors:** Alexa Gaucher-Holm, Christine Mulligan, Mary R. L’Abbé, Monique Potvin Kent, Lana Vanderlee

**Affiliations:** 1 École de nutrition, Centre Nutrition, santé et société (NUTRISS), Institut sur la nutrition et les aliments fonctionnels (INAF), Université Laval, 2440 Bd Hochelaga, Québec, QC G1V 0A6 Canada; 2 Department of Nutritional Sciences, University of Toronto, 1 King’s College Circle, Toronto, ON M5S 1A8 Canada; 3 School of Epidemiology and Public Health, University of Ottawa, 600 Peter Morand, Ottawa, ON K1G 5Z3 Canada

**Keywords:** Lobbying, Corporate political activity, Public health policy, Nutrition policy, Industry

## Abstract

**Background:**

The political activities of industry stakeholders must be understood to safeguard the development and implementation of effective public health policies.

**Methods:**

A quantitative descriptive study was performed using data from Canada’s Registry of Lobbyists to examine the frequency and governmental target of lobbying that occurred between various types of stakeholders (i.e., industry versus non-industry) and designated public office holders (DPOH) regarding Health Canada’s *Healthy Eating Strategy*, from September/2016 to January/2021. Initiatives of interest were revisions to Canada’s Food Guide, changes to the nutritional quality of the food supply, front-of-pack nutrition labelling and restrictions on food marketing to children.

**Results:**

The majority of registrants (88%), and corporations and organizations (90%) represented in lobbying registrations had industry ties. Industry-affiliated stakeholders were responsible for 86% of communications with DPOH, interacting more frequently with DPOH of all ranks, compared to non-industry stakeholders. Most organizations and corporations explicitly registered to lobby on the topic of marketing to children (60%), followed by Canada’s Food Guide (48%), front-of-pack nutrition labelling (44%), and the nutritional quality of the food supply (23%). The food and beverage industry, particularly the dairy industry, was the most active, accounting for the greatest number of lobbying registrations and communications, followed by the media and communication industry.

**Conclusions:**

Results suggest a strategic advantage of industry stakeholders in influencing Canadian policymakers. While some safeguards have been put in place, increased transparency would allow for a better understanding of industry discourse and help protect public health interests during the policy development process.

**Supplementary Information:**

The online version contains supplementary material available at 10.1186/s12992-022-00842-4.

## Introduction

Non-communicable diseases have been identified as a major threat to global health and sustainable development [1]. The prevention of these diseases will require international, and national-level interventions that address behavioral risk factors such as unhealthy dietary patterns [1, 2]. Substantial changes to food environments, defined as the set of sociocultural, economic, political and physical conditions which drive food choices [3, 4] will be necessary to modify dietary patterns.

**Fig. 1:**
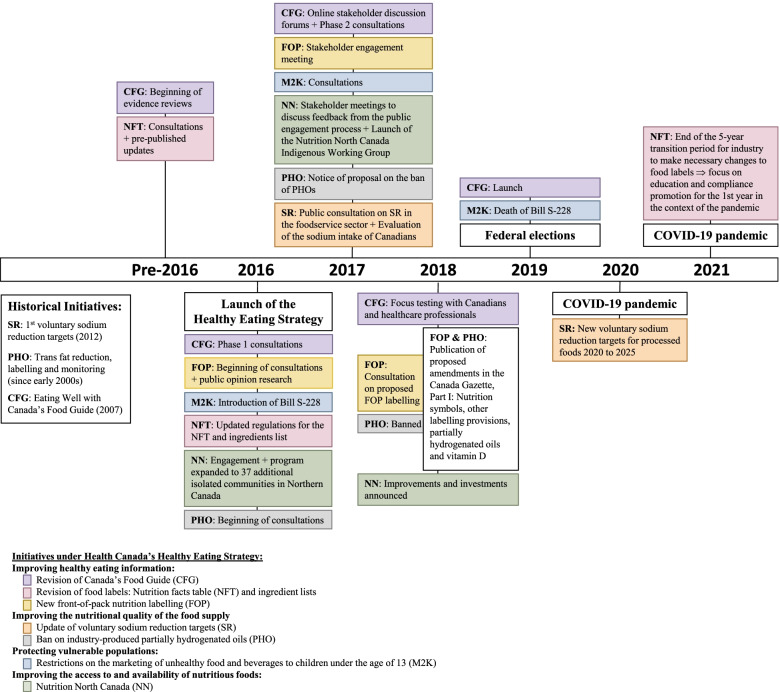
Timeline of Health Canada’s Healthy Eating Strategy [8–14]

In Canada, 63% of adults live with overweight or obesity [5], while 34% report living with at least one of the 5 major non-communicable diseases (e.g., diabetes, cardiovascular disease, and others) [6]. Moreover, dietary risk factors are significant within the Canadian population; 58% of Canadians over the age of 1 consume too much sodium [7], while 71% of Canadians over the age of 12 report consuming less than the recommended 5 portions of fruits and vegetables per day [6]. In 2016, the Government of Canada launched the Healthy Eating Strategy “to make it easier for Canadians to make the healthier choice” [8]. The Strategy is composed of a suite of 7 initiatives aimed at improving the Canadian food environment, the evolution of which is described in Fig. 1. Initiatives include the introduction of front-of-pack nutrition labelling, updates to the Nutrition Facts tables, the elimination of industry-produced partially hydrogenated oils, updated sodium reduction targets, restrictions on the marketing of unhealthy food and beverages to children, revisions to Canada’s Food Guide, and strategies to improve access to nutritious foods in northern communities (‘Nutrition North’).

Political will is necessary for governments to develop and implement policies aimed at improving food environments [15], and can be strongly influenced by industry stakeholders via corporate political activities. A growing body of literature is examining the political influence of powerful commercial actors. Globally, evidence indicates that the food and beverage industry uses a variety of tactics able to influence public health policy [16–21], ranging from coalition management, to the funding and dissemination of research and information that protects and/or promotes their interests, to political lobbying [21]. Industry stakeholders also have a strategic advantage in influencing policymakers, as they have been shown to have a greater number of ties with decision makers [22], a greater number of interactions with government officials [23, 24], and a greater number of communications with public office holders of higher power compared to non-industry stakeholders [23]. Political lobbying and the involvement of industry in the policy making process must be understood to minimize undue influence, protect public-health interests, and ensure that effective policies can be developed and implemented.

As part of Canada’s Regulatory Openness and Transparency Framework [25], Health Canada’s Healthy Eating Strategy incorporated a policy whereby communications and meetings between Health Canada and groups, organizations, industry and advocates (but not individuals representing themselves, other levels of government or foreign governments) are required to be publicly documented on the Meetings and Correspondence on Healthy Eating (MCHE) database [26]. These communications are not limited to lobbyists or lobbyist firms, but include general correspondence, roundtables, written letters or submissions from any individuals representing non-governmental groups or organizations, including industry, health-related nongovernmental organizations, and others. Details on meetings and correspondences between Health Canada and industry, and most other non-governmental stakeholders have been used in recent publications to describe lobbying towards Health Canada in the context of the Healthy Eating Strategy [24, 26]. However, less attention has been given to interactions that may have occurred with other government departments such as Agriculture and Agri-Food Canada (AAFC), who have considerable influence on Canadian food policies. Nonetheless, the activities of most paid lobbyists are publicly available in Canada on the Office of the Commissioner of Lobbying of Canada’s Registry of Lobbyists [27].

The objective of the current study was to quantify and describe the interactions recorded in the Registry of Lobbyists that occurred between industry and non-industry stakeholders, and public office holders from all institutions on topics related to the Healthy Eating Strategy.

## Methods

### Study design

A quantitative descriptive study was performed to examine the lobbying activities of industry and non-industry stakeholders surrounding the Healthy Eating Strategy. Initiatives of interest were the revision of Canada’s Food Guide, changes to the nutritional quality of the food supply (i.e., the elimination of industry-produced partially hydrogenated oils and the updated voluntary sodium reduction targets), restrictions on the marketing of unhealthy food and beverages to children under the age of 13 (hereafter “marketing to children”) and associated Bill S-228 (a bill that was proposed in September 2016 to restrict food and beverage marketing to children in Canada and ultimately failed to pass), and front-of-pack nutrition labelling; all of which correspond with the initiatives covered under Health Canada’s transparency policy [26]. Policies or actions related to Nutrition North (a government program to increase the access of nutritious foods in remote Northern communities) were excluded from this study as other government efforts beyond the Healthy Eating Strategy also address food security issues, such as Agriculture and Agri-Food Canada’s National Food Policy [28], which may have confounded the search. Moreover, updates to the Nutrition Facts table were excluded as consultations began in 2013 [29], and were finalized prior to the announcement of the Healthy Eating Strategy.

### Data sources

Canada’s *Lobbying Act* requires that individuals who are paid by an employer or client to lobby designated public office holders (DPOH) register their activities in an online database. Lobbyists can either be ‘consultant lobbyists’ who communicate with government on behalf of clients (i.e., corporations or organizations) and, thus, may simultaneously represent multiple organizations or corporations, or ‘in-house lobbyists’ who communicate with government on behalf of the organization or corporation by which they are employed. Lobbying Registrations and Communication Reports are publicly available on the Registry of Lobbyists [27]. Lobbying Registrations include information on the registrant (i.e., the person responsible for the submission and certification of lobbying registrations and communications), who they work for and represent, whether the corporation or organization they represent receives government funding, details on the subject(s) for which they lobbied (i.e., the subject matter details), who they plan to interact with (i.e., the government institution) and how (e.g., through written or oral communications), as well as the effective date of their registration. Registrations are required for all consultant lobbyists, whereas registrations are only required of in-house lobbyists if lobbying constitutes a significant part of the organization or corporation’s duties (i.e., at least 20% of an employee’s duties or the equivalent if performed by multiple employees) [30]. Volunteers and private citizens who lobby DPOH are not required to register [30].

Monthly Communication Reports describe oral and arranged communications (including phone conversations, meetings, and any other verbal communications) between lobbyists and DPOH [27]. They include information on the public office holders and government institutions which were lobbied, broad subject matters (e.g., Health, Agriculture, Consumer Issues, etc.), as well as the date of the communication. However, content details of a specific communication are not disclosed. Monthly Communication Reports are not required for interactions initiated by public office holders, unless these are related to financial benefits, or the awarding of contracts [30].

### Data extraction

Lobbying Registration files and Monthly Communication Reports were downloaded (as.csv files) on March 5^th^, 2021 [27]. Methods were adapted from previously published research [23].

### Lobbying Registrations

Data from the Registration files were extracted for lobbying registrations which became effective between September 1^st^, 2016, and January 31^st^, 2021, corresponding with the period of relevant policy activity (hereafter referred to as the “policy window”) for the Healthy Eating Strategy. Relevant registrations were first identified using keyword searches of the subject matter details (using Microsoft Excel). A list of keywords was established for each of the Healthy Eating Strategy initiatives included in this study and refined using the online Registry of Lobbyists, the Meetings and Correspondence on Healthy Eating database, and the Government of Canada’s website. Both French and English keywords were included. Broad terms were excluded from the search strategy to obtain a conservative estimate of lobbying activities. For instance, the terms “nutrition labelling” and “food labelling” were excluded from the search strategy as these may have referred to non-front-of-pack nutrition labelling such as health claims, ingredient lists, or the modernization of the Nutrition Facts table. All registrations related to the Healthy Eating Strategy and initiatives of interest were extracted. Various word combinations were used to filter through the excluded data to ensure that no relevant entries were missed.

Extracted registrations were scanned by AGH, and non-relevant or questionable registrations were removed on a case-by-case basis, in consultation with LV. LV conducted a secondary coding of the final included registrations (~ 8%, every tenth registration). A summary of the search strategy can be found in Appendix A (Table A.1). Extracted data included the registrant’s name and ID, as well as the name and ID of the corporation or organization represented in the lobbying registration.

**Table 1:** Number of registrants, and corporations/organizations represented in lobbying registrations, and their respective communications with DPOH

	**All**	**Industry** ***N* (%)**	**Non-Industry** ***N* (%)**
Number of registrants representing one or more corporations and/or organizations	170^1^	150 (88)^2^	21 (12)^2^
Number of corporations and organizations represented by one or more registrants	48	43 (90)	5 (10)
Number of communications	5197	4474 (86)	723 (14)

^1^ One registrant represented > 1 corporation/organization with industry ties and ≥ 1 with no industry ties. This registrant was accounted for in both industry and non-industry categories

^2^ Relative frequency is based on total of 170 registrants who lobbied about the Healthy Eating Strategy and/or at least one of its main initiatives

### Monthly Communications Reports

Communications which occurred between September 1^st^, 2016, and January 31^st^, 2021, were included in the study sample. Registrant IDs and corporation/organization names previously identified in the registration files were paired and used to extract communications which occurred between lobbyists and DPOH. Extracted data included the date of the communication, the government branch(es) and institution(s), as well as the title(s) and name of the DPOH(s) who participated in the interaction.

### Stakeholder classification

**Fig. 2:**
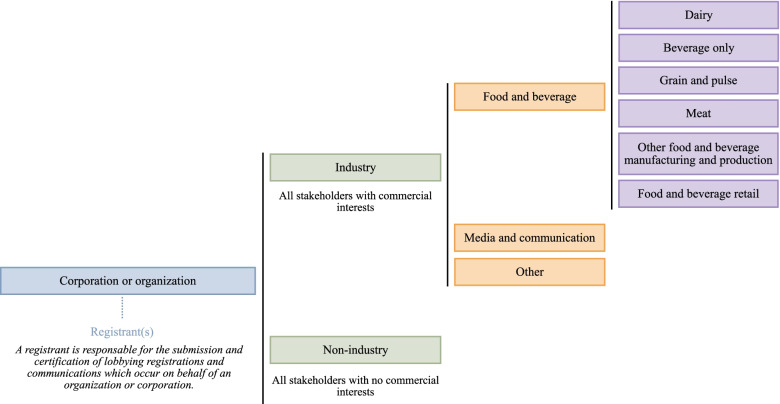
Decision tree used to categorize stakeholders (registrants and their associated corporations or organizations) who registered to lobby about the Healthy Eating Strategy

Stakeholders were categorized based on the commercial interest of the corporation or organization represented [23, 24]. Stakeholders with no perceived commercial interests, such as professional health organizations, were labelled as ‘non-industry’. Stakeholders with a perceived commercial interest or industry affiliation (including not-for-profit industry or commodity groups) were categorized as ‘industry’, and further categorized as described in Fig. 2.

### Ranking of designated public office holders

DPOH were categorized and ranked according to their title, branch and institution using a scheme adapted from Mulligan et al.’s recent publication (Appendix B, Table B.1) [23]. Targeted online searches of the DPOH were conducted when additional information was required to accurately rank the DPOH.

### Statistical analysis

Descriptive statistics were used to examine 1) the number of industry and non-industry registrants with lobbying registrations pertaining to the Healthy Eating Strategy, 2) the number of organizations and corporations represented by at least one registrant in lobbying registrations pertaining to the Healthy Eating Strategy, by stakeholder type (as depicted in Fig. 2) and healthy eating initiative, 3) the frequency of communications with DPOH by stakeholder type, 4) the frequency of communications with DPOH by DPOH rank, and 5) the frequency of interactions with government institutions by stakeholder type. Data extraction and statistical analyses were performed in RStudio (Version 1.4.1106) and Microsoft Excel software.

Ethics exemption was provided by Université Laval. Open access data can be retrieved from the Registry of Lobbyists (https://lobbycanada.gc.ca/en/open-data/) [27].

## Results

Between September 1st, 2016, and January 31st, 2021, 170 registrants registered to lobby on the topic of the Healthy Eating Strategy and/or at least one of the initiatives included in this study (Table 1). These registrants represented a total of 48 corporations, and organizations. Of the 170 registrants, 130 (76%) (representing a total of 44 (92%) corporations and organizations) filed communication reports (*n* = 5197) during the policy window for the Healthy Eating Strategy.

### Lobbying Registrations

Table 2 displays the type of organizations and corporations represented in lobbying registrations and initiatives of interest. Overall, 60% (*n* = 29) of corporations and organizations registered to lobby public office holders on the topic of food marketing to children, followed by 48% (*n* = 23) on Canada’s Food Guide, 44% (*n* = 21) on front-of-pack nutrition labelling and 23% (*n* = 11) on the nutritional quality of the food supply.

**Table 2:** Number of industry- and non-industry- affiliated organizations and corporations^1^ listed in lobbying registrations overall and by initiative

**Number of organizations and corporations by initiative (*n*)**	**Industry ** ***n*** **(%)**										**Non-Industry ** ***n*** **(%)**
	**All**	**Communication and media**	**Food and beverage**							**Other**	**All**
			**All**	**Beverage only**	**Dairy**	**Grain and pulse**	**Meat**	**Other food and beverage manufacturing and production**	**Food and beverage retail**		
**Nutritional quality of the food supply (*n* = 11)**	8 (73)	0 (0)	8 (73)	0 (0)	1 (9)	0 (0)	1 (9)	5 (45)	1 (9)	0 (0)	3 (27)
**Canada’s Food Guide (*n* = 23)**	19 (83)	1 (4)	18 (78)	3 (13)	8 (35)	1 (4)	2 (9)	4 (17)	0 (0)	0 (0)	4 (17)
**Front-of-pack labelling (*n* = 21)**	18 (86)	1 (5)	17 (81)	2 (10)	7 (33)	0 (0)	3 (14)	5 (24)	0 (0)	0 (0)	3 (14)
**Marketing to children (*n* = 29)**	25 (86)	7 (24)	16 (55)	3 (10)	3 (10)	2 (7)	1 (3)	4 (14)	3 (10)	2 (7)	4 (14)
**Healthy Eating Strategy (overall)**^**2**^ **(*N* = 48)**	43 (90)	9 (19)	32 (67)	3 (6)	11 (23)	3 (6)	3 (6)	8 (17)	4 (8)	2 (4)	5 (10)

^1^ Includes all organizations and corporations represented by at least 1 registrant in lobbying registrations

^2^ Includes general entries about the Healthy Eating Strategy and entries specific to any of the initiatives included in this study

Most corporations and organizations (90%, *n* = 43) who registered to lobby were perceived as having industry ties. Of the industry-affiliated organizations and corporations, 16 (37%) were companies (e.g., Coca Cola Ltd), while 26 (60%) were industry or commodity groups or associations (e.g., The Canadian Beverage Association, and Dairy Farmers of Canada), and 1 (2%) was a not-for-profit organization working with industry (e.g., GS1 Canada). Industry-affiliated corporations and organizations most frequently registered to lobby on the topic of marketing to children (58%, *n* = 25), followed by Canada’s Food Guide (44%, *n* = 19), front-of-pack nutrition labelling (42%, *n* = 18) and, lastly, the nutritional quality of the food supply (19%, *n* = 8).

Of the 10% (*n* = 5) of organizations/corporations with no industry affiliations, most (80%) registered to lobby on the topic of Canada’s Food Guide and marketing to children, followed by the nutritional quality of the food supply (60%), and front-of-pack nutrition labelling (60%).

### Communications with designated public office holders

A total of 5197 communications occurred between DPOH and individuals registered to lobby on the topic of the Healthy Eating Strategy on behalf of a specific corporation or organization between September 2016, and January 2021. Industry-affiliated stakeholders accounted for the majority of communications with DPOH (86%, *n* = 4474). Industry and commodity groups or associations were responsible for more than half of all communications (53%; *n* = 2732), while individual industry-affiliated organizations and companies were responsible for a third (34%, *n* = 1742) of all communications.

As shown in Table 3, the food and beverage industry was responsible for in the majority (66%) of communications with DPOH. The dairy industry was the most active food and beverage subcategory (24%, *n* = 1262), followed by the “other food and beverage manufacturing and production” industry (14%, *n* = 726), which grouped a variety of processed/packaged food companies (i.e., confectionery, bakery, and pizza), as well as general food and beverage, processing, and consumer product associations. Media and communication industry-affiliated lobbyists were the second most active in communicating with DPOH (20%, *n* = 1024).

**Table 3:** Communications between DPOH and stakeholders who registered to lobby about the Healthy Eating Strategy

	**All ** ***N* (%)**	**Industry** ***n* (%)**										**Non-Industry ** ***n* (%)**
		**All**	**Communication and media**	**Food and Beverage**							**Other**	**All**
				**All**	**Beverage Only**	**Dairy**	**Grain and pulse**	**Meat**	**Other food and beverage manufacturing and production**	**Food and beverage retail**		
**Number of Communications**	5197 (100)	4474 (86)	1024 (20)	3424 (66)	547 (11)	1262 (24)	265 (5)	365 (7)	726 (14)	259 (5)	26 (1)	723 (14)

As shown in Table 4, industry stakeholders interacted more often with DPOH of any rank, compared to non-industry stakeholders. Both industry- and non-industry-affiliated stakeholders most frequently lobbied Members of Parliament, Senators and their staff, followed by ministerial staff.

**Table 4:** Number of DPOH^1^ who participated in communications with industry- and non-industry-affiliated stakeholders by rank (*N* = 7150).^2^

Rank	Communications with industry stakeholders (*n* = 6345) *n* (%)	Communications with non-industry stakeholders (*n* = 805) *n* (%)
**Parliamentarians and their staff**	**4807 (67)**	**719 (10)**
Prime Minister’s Office	321 (4)	17 (< 1)
Ministers and Parliamentary Secretaries	406 (6)	35 < 1)
Ministerial staff	1561 (22)	99 (1)
Members of Parliament, Senators, and their staff	2519 (35)	568 (8)
**Civil Servants**	**1538 (22)**	**86 (1)**
Privy Council Office	39 (1)	5 (< 1)
Deputy Ministers	249 (3)	6 (< 1)
Assistant Deputy Ministers or the functional head of an agency or crown corporation	570 (8)	24 (< 1)
Other government officials	680 (10)	51 (1)

^1^ A designated public office holder was accounted for more than once if they participated in multiple communications

^2^ Certain communications occurred with multiple designated public office holders simultaneously, and each individual was included in the sample denominator

The most frequently lobbied institution overall was the House of Commons (the lower chamber of parliament that consists of elected officials) (*n* = 2908, 41%), followed by Agriculture and Agri-Food (AAFC) (*n* = 843, 12%), Health Canada (HC) (*n* = 559, 8%), and Innovation, Science and Economic Development Canada (ISED) (*n* = 552, 8%) (Appendix C, Table C.1). Industry-affiliated stakeholders interacted most with DPOH from the House of Commons (*n* = 2429), followed by AAFC (*n* = 831), and ISED (*n* = 548), while non-industry stakeholders interacted most with DPOH from the House of Commons (*n* = 479), followed by the Senate of Canada (*n* = 105), and HC (*n* = 85).

## Discussion

### Lobbying registrations by stakeholder type and healthy eating initiative

The voice of industry stakeholders dominated the lobbying landscape during the policy window for the Healthy Eating Strategy, as demonstrated by the proportion of industry-affiliated registrants (88%), and corporations and organizations (90%) in lobbying registrations and the number of interactions between industry-affiliated stakeholders and DPOH (86%). The results of the study show proportionately more interactions with industry stakeholders compared to a recent publication (86% versus 56%) that looked into interactions with Health Canada on the topic of the Healthy Eating Strategy using the MCHE database over a shorter time period [24]. Moreover, the results are in line with those of a recent publication which examined lobbying in the context of marketing to children in Canada and the failed Bill S-228 on marketing restrictions [23]. The proportionately small number of non-industry stakeholders lobbying on the topic of the Healthy Eating Strategy is cause for concern given that they advocate for public health, rather than economic interests.

Healthy eating initiatives of interest in lobbying registrations varied by stakeholder type. The vast majority (86%) of organizations and corporations represented in lobbying registrations pertaining to marketing to children had industry-affiliations. In addition to the food and beverage industry, the media and communication industry was also actively involved in lobbying activities surrounding marketing to children. This is unsurprising, given that these industries may see marketing restrictions as a direct threat to their commercial and economic interests. Moreover, this highlights corporate players beyond the food and beverage industry that have vested interests in nutrition policies, and who should not be overlooked when implementing measures to safeguard the development of public policies. Although impossible to determine the impact of lobbying in this study, these results suggest that industry viewpoints were most prominent during the time when Bill S-228 that proposed marketing restrictions was being considered, which may have played a role in its eventual demise in 2019 [14, 31]. These results are also in line with Mulligan et al.’s recent publication which included data from both the Registry of Lobbyists and the MCHE database [23].

Canada’s Food Guide also received considerable attention from the food and beverage industry, particularly the dairy industry. Partial lobbying restrictions were put in place during the revision of the Food Guide to minimize potential conflict of interest with regard to Health Canada. In fact, officials from Health Canada’s Office of Nutrition Policy and Promotion, the office responsible for the revision of Canada’s Food Guide, did not interact with stakeholders from the food and beverage industry during the development process [12]. Therefore, lobbying may have occurred, but only with other government offices or departments outside of the Office of Nutrition Policy and Promotion. This is demonstrated by the considerable number of industries that directly mentioned the Food Guide in their lobbying registrations. Although impossible to measure the impact of lobbying in this study, significant changes to the most recent version of Canada’s Food Guide suggest that these safeguards may have been effective. For instance, despite heavy lobbying from the dairy industry, dairy was removed as a food group, and placed within the general category of protein foods in the revised guide [32] reflecting current scientific evidence rather than economic interests.

The majority of corporations and organizations registered to lobby on the topic of front-of-pack labelling (86%) had industry affiliations, and most were from the food and beverage industry, particularly the dairy and “other food and beverage” industries. In fact, there was considerable concern voiced from the dairy and beverage industries that this type of labelling would unreasonably penalize their products [33–36]. Remarkably, less than half (44%, *n* = 21) of all corporations and organizations explicitly registered to lobby on the topic of front-of-pack nutrition labelling. Nonetheless, using the MCHE database, Vandenbrink et al. showed that in interactions between Health Canada and industry stakeholders, the most frequently discussed topic was front-of-pack labelling [24]. The differences in results may be partly explained by the purposeful exclusion of broad terms such as “nutrition labelling” from the search strategy in the present study, which provides a conservative estimate of lobbying for this topic in particular, as well as the different timing of the studies. Moreover, the present study identifies who registered to lobby on the topic of front-of-pack labelling, rather than instances of communication specific to front-of-pack nutrition labelling; these may have been more numerous, but this could not be examined using the current dataset.

Less than a quarter of corporations and organizations registered to lobby on the topic of the nutritional quality of the food supply, a policy area which would mostly affect the processed and packaged food industry. In fact, most of the organizations and corporations fell within the “other food and beverage production and manufacturing” category. The small number of organizations and corporations who registered to lobby on this topic may be explained by the voluntary nature of the sodium reduction targets [37], the historical nature of the sodium and trans-fat initiatives (which began in the early 2000s [38, 39]), and the search strategy, which purposefully excluded lobbying registrations referring to the historical trans-fat and sodium reduction initiatives (Supplemental Table S1) to obtain a conservative estimate of lobbying activities.

While this study is not able to assess the causal effect of lobbying, these lobbying data may reflect the policy pathway and current status of the major policies within the Strategy: the only initiative with extensive safeguards during the policy development process (i.e., Canada’s Food Guide) resulted in significant changes and successful implementation. On the other hand, the policy which received the greatest amount of attention from industry (i.e., marketing to children) resulted in failed policy implementation. Moreover, front-of-pack labelling, for which lobbying has occurred and no additional safeguards put in place, has not been implemented as of the date of publication. On the other hand, the initiatives pertaining to the nutritional quality of the food supply received only some lobbying attention which may be related to there having been minimal action immediately required on behalf of the food industry (e.g., given the voluntary nature of the sodium reduction initiative).

### Communications by stakeholder type and public office holder ranking

Industry-affiliated stakeholders were shown to have more interactions with DPOH of all ranks, including those in greater positions of power, compared to non-industry stakeholders. In fact, industry-affiliated stakeholders were responsible for 14 times more communications with DPOH of the two highest ranks (within “Parliamentarians and their staff”) compared to non-industry stakeholders, suggesting an uneven playing field and strategic advantage of industry in influencing government officials. Access to policy makers in positions of power has been studied in Australia, where food industry stakeholders were in fact shown to have strategic relationships and access points to decision makers providing them with a greater capacity to lobby and influence nutrition policy compared to other professionals [22].

### Limitations

Multiple limitations relate to the data available on the Registry of Lobbyists. Firstly, lobbying activities were likely underestimated as the *Lobbying Act* does not require registrations from private citizens, volunteers, and in-house lobbyists when lobbying does not represent a significant part of their organization’s or corporation’s duties [30]. Secondly, as the content of each specific communication is not disclosed, communications may have been about any subject matter declared by a registrant who registered to lobby on the topic of the Healthy Eating Strategy on behalf of an organization or corporation. To address this, the current study analyzed both registrations and instances of communication; the results should be considered in light of this limitation. Moreover, communication data from the Registry, which was used to rank DPOH, was at times incomplete (e.g., the branch unit was missing from the entry), requiring assumptions related to the government official’s position. For example, DPOH were assumed to have represented the same branch unit if they were present in multiple communications as long as their title and institution remained the same. Targeted online searches were conducted when necessary (i.e., if the missing information was required to rank the DPOH). In addition, certain DPOH held more than one position at a point in time, and only one of the positions was entered into the registry. Therefore, for those with dual roles (such as when a Minister is also a member of Parliament, two distinct roles within our evaluation scheme), ranking was based on the title indicated in the specific communication. A recent report from the Commissioner of Lobbying offers preliminary recommendations to improve the *Lobbying Act* in Canada [40]. These recommendations would address numerous limitations described in this study, such as amending the threshold for in-house lobbying registrations and expanding reporting requirements for communication reports.

Finally, the search strategy used to identify lobbying activities in the context of the Healthy Eating Strategy likely presents a conservative estimate of lobbying instances. Firstly, initiatives were solely accounted for if explicitly named by the registrant. Moreover, general terms such as ‘labelling’ and ‘nutrition’ were omitted as non-specific to the Healthy Eating Strategy, even though they may have included discussion about associated policies and initiatives. Second, two initiatives (Nutrition North Canada and changes to the Nutrition Facts tables) were excluded from the study.

## Conclusion

The sheer volume of registrants, corporations, and organizations with industry affiliations registered to lobby on the topic of the Healthy Eating Strategy and their communications with all levels of government, particularly those of higher power, suggest a major strategic advantage in influencing policymakers. This underscores the need for increased capacity and support for non-industry stakeholders to equally have their voices heard among government policymakers in discussions related to key food and nutrition policies in Canada (e.g., through the funding of knowledge translation efforts or civil-academic-NGO collaborations to provide key evidence to policy advocates).

While Canada has demonstrated some leadership in political transparency, including its publicly accessible and easily downloadable dataset of lobbying activities [27], its revolving-door policy for public servants acting as lobbyists for up to 5 years after their governmental position ends [30], limits on election campaign contributions and publicly available database of contributions [41], as well as novel openness and transparency policies in policymaking related to food [26], these policies can be further strengthened to enable improved monitoring of these activities. In fact, the analysis of data from the Registry of Lobbyists demonstrates the need for improvements to Canada’s *Lobbying Act*. For instance, requiring the disclosure of the contents of communications on the Registry of Lobbyists and removing the thresholds for in-house lobbyists could greatly increase transparency surrounding the development and implementation of public health nutrition policies in Canada, as well as the level and type of influence that external groups have on the development of these policies.

This study provides an important case study highlighting the involvement of industry in the policy-making process and supports government action to address the transparency, disclosure, and management of this corporate political activity. Considering the urgent need for public policy to address the underlying causes of non-communicable disease, additional safeguards should be considered to prevent undue interference from stakeholders with commercial interests during the policy-development process.

## Supplementary Information

**Additional file 1.** 

## Data Availability

The datasets supporting the conclusions of this article are available in the Office of the Commissioner of Lobbying of Canada’s Registry of Lobbyists, https://lobbycanada.gc.ca/en/open-data/ (27).

## Abbreviations

AAFC: Agriculture and Agri-Food Canada
CFG: Canada’s Food Guide
DPOH: Designated public office holder
FOP: Front-of-pack nutrition labelling
HC: Health Canada
ISED: Innovation, Science and Economic Development Canada
MCHE: Meetings and Correspondence on Healthy Eating
M2K: Marketing of unhealthy food and beverages to children
NFT: Nutrition facts table
NN: Nutrition North Canada
PHO: Partially hydrogenated oils
SR: Sodium reduction
