# Supplementary material for: Lobbying and nutrition policy in Canada: a quantitative descriptive study on stakeholder interactions with government officials in the context of Health Canada’s Healthy Eating Strategy
Source: Global Health. 2022 May 26;18:54. doi: 10.1186/s12992-022-00842-4 (PMC9137051; doi:10.1186/s12992-022-00842-4)
Supplement: Supplementary file 1 — Additional file 1. [file 12992_2022_842_MOESM1_ESM.docx]

# **Appendix A.**

**Table A.1. Search strategy used to identify relevant lobbying registrations pertaining to the Healthy Eating Strategy**

|  | **Healthy Eating Strategy** | **Nutritional quality of the food supply** | **Canada’s Food Guide** | **Front-of pack labelling** | **Marketing to children** |
| --- | --- | --- | --- | --- | --- |
| **Keywords** | - Healthy Eating Strategy - Stratégie de Santé Canada en matière de saine alimentation - Stratégie en matière de saine alimentation - Eating Healthy Strategy - Health Eating Strategy - Food and Drug Act (Nutrition Symbols, Other Labelling Provisions, Partially Hydrogenated Oils and Vitamin D) - Food and Drug Regulations (Nutrition Symbols, Other Labelling Provisions, Partially Hydrogenated Oils and Vitamin D) | - Sodium - Partially hydrogenated oils - Trans fat - Huiles partiellement hydrogénées - Gras trans | - Food Guide - Guide alimentaire - Nutritional guidelines - Nutrition guidelines | - Symboles nutritionnels - Nutrition symbols - Front-of-pack - Front of pack | - Marketing to kids - Marketing to children - Marketing of food - Advertising restrictions - Marketing of unhealthy food and beverages - Advertising directed at children - Advertising to children - Food advertising - S-228   *All entries with “Child Health Protection Act” also referenced Bill S-228*. |
| **Additional Specifications** | All entries related to the 4 initiatives of interest were added to this category.  *Broad terms such as ‘’Nutrition initiatives under Health Canada’’ were excluded as they were deemed unspecific and may have referred to initiatives beyond those of the Healthy Eating Strategy.* | The following entries were removed, unless a second statement clearly identified the sodium or trans-fat initiatives under the Healthy Eating Strategy (these entries were considered to be historical):   - Sodium reduction strategy - Trans fat task force - Trans fat regulatory framework   The following stray entry was removed:   - Sodium of Potash production   The following entries were removed and included under ‘’Healthy Eating Strategy’’ unless a second sentence clearly identified nutritional quality initiatives:   - Food and Drug Regulations (Nutrition Symbols, Other Labelling Provisions, Partially Hydrogenated Oils and Vitamin D) - Food and Drug Act (Nutrition Symbols, Other Labelling Provisions, Partially Hydrogenated Oils and Vitamin D) - Règlement modifiant certains règlements pris en vertu de la Loi sur les aliments et drogues (symboles nutritionnels, autres dispositions d’étiquetage, huiles partiellement hydrogénées et vitamine D) | Passive entries about Canada’s Food Guide were removed (i.e., if the food guide was used to support an argument).  e.g., ‘*’As per Canada's food guide, we encourage the public to consume milk to meet the daily dairy requirements of important vitamins and minerals.*’’ | Entries containing (Label AND (sodium OR fat OR sugar)) were checked (in both French and English) and included if relevant and clearly not in the context of the labelling modernization initiative (e.g., ‘’Introduce improved sugar and sodium labelling on food products’’).  The following entries were removed and included under ‘’Healthy Eating Strategy’’ unless a second sentence clearly identified front-of-pack labelling:   - Food and Drug Regulations (Nutrition Symbols, Other Labelling Provisions, Partially Hydrogenated Oils and Vitamin D) - Food and Drug Act (Nutrition Symbols, Other Labelling Provisions, Partially Hydrogenated Oils and Vitamin D) - Règlement modifiant certains règlements pris en vertu de la Loi sur les aliments et drogues (symboles nutritionnels, autres dispositions d’étiquetage, huiles partiellement hydrogénées et vitamine D) | Entries containing ((kids OR children) AND food), or ((kids OR children) AND (advertising OR marketing)) were checked.  Entries about marketing which were NOT clearly in the context of the Healthy Eating Strategy (M2K) (e.g., children's advertising initiative) were removed.  No relevant French keywords were found to have been used by stakeholders in lobbying registrations (e.g. “Restrictions de la publicité”, “Publicité destinée aux enfants”, “Publicité sur les aliments”…) |

# **Appendix B.**

**Table B.1. Designated public office holder ranking and categorization scheme^1^**

| **Categories and ranks** | **Description** |
| --- | --- |
| **Parliamentarians and their staff** | |
| Prime Minister’s Office | - The Prime Minister and anyone registered with the Prime Minister’s Office - The Deputy Prime Minister and anyone registered with the Deputy Prime Minister |
| Ministers and Parliamentary Secretaries | - Ministers (including the Minister of Democratic Institutions) - Parliamentary secretaries |
| Ministerial staff | - Anyone working directly for a minister or parliamentary secretary (e.g., policy advisor, chief of staff, communication advisor to a minister) |
| Members of Parliament, Senators and their staff | - Members of parliament - Shadow ministers - Senators - Anyone registered with the House of Commons or the Senate of Canada |
| **Civil Servants** | |
| Privy Council Office | - Anyone registered with the Privy Council Office (unless they are a minister or associated to a Minister’s office) |
| Deputy Minister | - Deputy Ministers - Associate Deputy Ministers |
| Assistant Deputy Minister ***or***  The functional head of an agency or crown corporation | - Assistant deputy ministers - Acting and associate assistant deputy minister   **Functional heads**:   - Canadian Dairy Commission (CDC): Chief executive officer (CEO), and chairperson - Canadian Food Inspection Agency (CFIA) : President - Competition Bureau (COBU) : Commissioner of competition - Public Health Agency of Canada (PHAC) : President, and chief public health officer - Canadian Grain Commission (CGC): Chief Commissioner - Canadian Radio Television and Telecommunications Commission (CRTC): Chairperson, and CEO - Treasury Board of Canada Secretariat (TBS): President - Stats Canada: Chief statistician of Canada - Communications Security Establishment Canada (CSEC): Head/associate head |
| Other government officials | - Any civil servant registered with a government institution other than the House of Commons, or the Senate of Canada not previously accounted for in a higher rank (e.g., directors, vice presidents, chief strategy officers, chief trade negotiator, chief operating officer, …) |

^1^ Table adapted from Mulligan et al. (23).

# **Appendix C.**

**Supplementary table C.1. Number of times government institutions^1^ were represented by a DPOH (N=7150) in communications with industry and non-industry stakeholders**

| Institution | All  N (%) | Industry  N (%) | Non-industry  N (%) |
| --- | --- | --- | --- |
| House of Commons | 2908 (41) | 2429 (34) | 479 (7) |
| Agriculture and Agri-Food Canada (AAFC) | 843 (12) | 831 (12) | 12 (<1) |
| Health Canada (HC) | 559 (8) | 474 (7) | 85 (1) |
| Innovation, Science and Economic Development Canada (ISED) | 552 (8) | 548 (8) | 4 (<1) |
| Senate of Canada | 379 (5) | 274 (4) | 105 (1) |
| Global Affairs Canada (GAC) | 376 (5) | 376 (5) | 0 (0) |
| Prime Minister's Office (PMO) | 327 (5) | 311 (4) | 16 (<1) |
| Canadian Heritage (PCH) | 253 (4) | 249 (3) | 4 (<1) |
| Finance Canada (FIN) | 204 (3) | 188 (3) | 16 (<1) |
| Treasury Board of Canada Secretariat (TBS) | 131 (2) | 108 (2) | 23 (<1) |
| Canadian Food Inspection Agency (CFIA) | 106 (1) | 106 (1) | 0 (0) |
| Privy Council Office (PCO) | 66 (1) | 61 (1) | 5 (<1) |
| Employment and Social Development Canada (ESDC) | 63 (1) | 45 (1) | 18 (<1) |
| Canadian Radio-television and Telecommunications Commission (CRTC) | 53 (1) | 53 (1) | 0 (0) |
| Environment and Climate Change Canada (ECCC) | 48 (1) | 47 (1) | 1 (<1) |
| Other government institutions^1^ | 282 (4) | 245 (3) | 37 (<1) |

^1^ Government institutions represented <0.5% of the time.
